# A psychrotolerant strain of *Serratia marcescens* (MTCC 4822) produces laccase at wide temperature and pH range

**DOI:** 10.1186/s13568-014-0092-1

**Published:** 2015-01-16

**Authors:** Gaurav Singh Kaira, Kusum Dhakar, Anita Pandey

**Affiliations:** Biotechnological Applications, G. B. Pant Institute of Himalayan Environment and Development, Kosi-Katarmal, Almora, 263 643 Uttarakhand India

**Keywords:** Laccase, Psychrotolerant, *Serratia marcescens*, Organic solvents, Indian Himalayan Region (IHR)

## Abstract

A psychrotolerant bacterial strain of *Serratia marcescens*, originally isolated from a glacial site in Indian Himalayan Region (IHR), has been investigated for laccase production under different culture conditions. The bacterial strain was found to grow between 4 to 45°C (opt. 25°C) and 3 to 14 pH (opt. 5 pH) on prescribed growth medium, coinciding with production of laccase in laccase producing medium. However, the production of laccase was more consistent toward alkaline pH. Laccase enzyme was partially purified using gel filtration chromatography. The molecular mass of laccase was determined ~53 kDa on native PAGE. The Km and Vmax values were determined to be 0.10 mM and 50.00 μM min^−1^, respectively, with ABTS. Inoculum size (4.0% v/v at 1.5 O.D.) resulted in significantly higher production of laccase. Carbon and nitrogen sources also affected the laccase production significantly. All the carbon sources enhanced laccase production, xylose being the best enhancer (P < 0.01). Among nitrogen sources, organic sources were found to act as inhibitors (P < 0.01), and among the in-organic sources only sodium nitrate enhanced the laccase production. Low molecular weight organic solvents significantly (P < 0.01) enhanced laccase production up to 24 h of incubation with a decline in later incubation period. Production of laccase by the psychrotolerant bacterium in wide range of temperature and pH is likely to have inference in biotechnological processes.

## Introduction

Laccases (benzenediol: oxygen oxidoreductase, EC 1.10.3.2) are multicopper oxidases that catalyze oxidation of an extensive array of recalcitrant phenolic and nonphenolic compounds with simultaneous reduction of molecular oxygen to water (Rivera-Hoyos et al. 2013). Catalytic diversity of laccases are exploited for a number of industrial and environmental applications such as dye effluent decolorization, bio-bleaching, xenobiotics bioremediation, biosensors and food industry (Couto and Herrera 2006). Despite the naturally evolved recalcitrance of lignin, the enzymatic approach to depolymerization also stretched out to eukaryotic and prokaryotic domains of life form. In nature, laccases are found to be widely distributed among plants, fungi, insects and bacteria (Alexandre and Zhulin 2000; Madhavi and Lele 2009).

Laccases have mainly been explored from fungal sources for their various applications, especially towards the lignin depolymerization. However, the potential of fungal laccases are limited by the metabolic pH and salt intolerance. Generally, the fungal laccases are known with their optimum activity between pH 2.0-4.0, that can be attributed to their growth adaptation towards acidic pH. Consequently, a need to increase this active pH range for laccase production through molecular engineering is being realized (Torres-Salas et al. 2013). Besides, relatively slow growth is another limitation towards the laccase production from fungal sources. In view of these limitations, laccase production from alternate sources, such as bacteria, is getting attention. Bacteria possess merits in terms of their ability to adapt to extreme physico-chemical conditions and feasibility for amenable recombinant expression (Joseph et al. 2014; Sharma et al. 2007; Sondhi et al. 2014). Industrial scale-up of bacterial laccases is still riddled due to its extracellular location and tolerance to extreme culture conditions. Being an extracellular enzyme, it has to tolerate the adverse environmental conditions pertaining to extremozyme category. To overcome all these limitations there is a need for identification of novel bacterial strains possessing the ability to produce extremozymes under extreme conditions. The tolerance towards the wide range of pH and temperature is an emblem of microbial strains making them potential candidates for various biotechnological processes.

*Serratia marcescens*, a gram negative bacterium from the family *Enterobacteriaceae*, has been recognized for its various applications in degradation (Manangeeswaran et al. 2007; Perestelo et al. 1994; Verma and Madamwar 2003). *S. marcescens* H30 has recently been studied for its potential in formation of one of the bulk chemicals (2,3-Butanediola) of biotechnological as well as industrial relevance (Zhang et al. 2014). However, studies based on its optimization for production of laccase under various physico-chemical and nutritional conditions are still negligible. Psychrotolerant strains of *S. marcescens* have been frequently isolated from high altitudes, including glacial sites, in the author's laboratory. The focus of the present study is on the production, purification and characterization of laccase from a psychrotolerant strain of *S. marcescens* (MTCC 4822) at a wide pH and temperature range.

## Material and methods

### Study site and the bacterium

The bacterium was isolated from the soil collected from a glacial site in the IHR (altitude: 3040 m above mean sea level; state Uttarakhand). Bacterial isolation was carried out following 10-fold serial dilutions and pour-plate method on Tryptone Yeast extract (TY) agar. The pure culture was maintained on TY agar slants at 4°C and in 10% glycerol at −20°C. All the experiments were conducted after raising fresh cultures. The bacterium was characterized on the basis of cultural (oxygen requirement), morphological (colony morphology and pigmentation), microscopic (Gram reaction and cell morphology), biochemical (utilization of carbon sources and enzyme activity), physiological (temperature, pH and salt tolerance) and molecular (16S rRNA gene sequence) methods. The bacterial isolate and its nucleotide sequence have been deposited in the Microbial Type Culture Collection and Gene Bank (IMTECH), Chandigarh, India and NCBI, Bathesda, Maryland, US, respectively.

### Inoculum preparation and laccase activity

A distinct bacterial colony from 24 h old agar plate culture was aseptically inoculated in 250 ml Erlenmeyer flask containing 50 ml modified Kirk and Farrell (1987) broth medium (Dhakar and Pandey 2013) pH 4.50 ± 0.5. The mother culture was raised at 25°C for 24 h under static and aerobic conditions. 1.0% v/v (O.D._600nm_ 1.50 ± 0.25) inoculum from mother culture was inoculated in 20 ml of the respective medium and incubated in the static condition. Following incubation, broth culture was centrifuged at 8000 rpm at 4°C for 10 min, the supernatant was treated as crude enzyme. Laccase activity was determined by ABTS (2, 2'-azino-bis (3-ethylbenzothiazoline-6-sulphonic acid)). Reaction mixture contained 0.10 M citrate- phosphate buffer (pH 2.5), ABTS (2 mM) and crude enzyme. Following two minutes of incubation at room temperature, activity was measured at 420 nm (Han et al. 2005). Enzyme unit was defined as 1 μM of ABTS oxidized per min under above standard assay conditions.

The growth curve on Kirk and Farrell medium with the laccase production up to 48 h (every 6 h) of incubation was recorded at 25°C.

### Partial purification and characterization of laccase

Bacterial culture, grown at 25°C for 48 h, was centrifuged at 8000 rpm at 4°C for 10 min. Supernatant was used for further process. Chilled acetone was added in 1:1 volume and kept at −20°C, overnight. Then, it was centrifuged at 12000 rpm for 20 min at 4°C. The pellet was re-dissolved in citrate- phosphate buffer (pH 2.6). The sample was further purified using gel filtration chromatography. Column (10 X 1 cm) was prepared using Sephadex G- 75 (Sigma). It was equilibrated with the citrate-phosphate buffer (pH 2.5). The fractions were collected every 3 min; the flow rate of the column was 1 mL min^−1^. Enzyme activity (ABTS assay) and total protein concentration by Lowry’s method was estimated at every step of the purification. The partially purified sample was used for the determination of Km and Vmax. Enzyme activity was determined with the different concentration of ABTS (0.05 mM to 1.00 mM). Lineweaver Burk plot was drawn between 1/(V μM min^−1^) and 1/([S] mM) concentration. Km and Vmax values were calculated using the graph. Molecular weight of the enzyme was determined using native polyacrylamide gel consisting 12.50% separating and 4% of stacking gel. After electrophoresis, gel was incubated in 0.10% ABTS in citrate- phosphate buffer (pH 2.5) for 20 min at room temperature. Green color band due to the oxidation of ABTS appeared on the gel. Protein marker was also run with the sample to determine the approximate molecular mass of the enzyme.

### Effect of the inoculum size, the physico-chemical and nutritional conditions, and the organic solvents on laccase production

#### The inoculum size

The laccase producing medium was inoculated with the different inoculum size ranging from 0.125 to 5.00% (0.125, 0.25, 0.50, 1.00, 2.00, 4.00, 5.00%), separately. The broth culture was incubated at 25°C in static conditions and observations were taken at 24 and 48 h of incubation.

#### The physico-chemical and nutritional conditions

In physico-chemical conditions, the laccase production was studied at different temperatures ranged from 5–45°C (at an interval of 10°C) and at the pH range from 3–13 (at an interval of 2 units). 1 N HCl and 1 N NaOH were used for maintaining the initial medium pH. For nutritional conditions, eight carbon (glucose, fructose, maltose, xylose, galactose, sucrose, starch and cellulose) and eight nitrogen (ammonium nitrate, ammonium chloride, ammonium sulfate, yeast, casein, peptone, urea, sodium nitrate) sources (0.20%) were supplemented in the medium, separately. Glucose and ammonium nitrate, being the ingredients in the original medium, were considered as control.

#### The organic solvents

Five low molecular weight organic solvents, namely methanol, ethanol, acetone, iso-propanol and iso-amyl alcohol were added to the medium in four concentrations (0.50, 1.00, 1.50 and 2.00%), separately, after 12 h of incubation.

All these experiments were performed at 25°C (excluding effect of temperature) for 48 h under static conditions.

### Statistical analysis

All the experiments were done in triplicate. Results were analyzed using one way ANOVA and two way repeated measures with Post-hoc Tukey’s test. The significance level was P < 0.05 and P < 0.01.

## Results

### The bacterium and the growth curve

The bacterium developed blood red colonies on TY agar plates and showed short and single Gram negative rods under microscope. In carbon utilization experiments, it could utilize 7 out of 12 tested sugars. It was positive for catalase and oxidase, and negative for lactose and starch hydrolysis. The bacterium was aerobic in nature and could grow between 4 to 45°C (opt. 25°C) and 3 to 14 pH (opt. 5–7 pH). The growth was observed restricted and without pigment beyond 35°C. It could tolerate salt up to 20%. In molecular characterization, it showed 98.8% similarity to *Serratia marcescens* N4-5. The bacterium has been allotted accession number (MTCC 4822) by IMTECH, Chandigarh, India and the nucleotide sequence accession number EF 462913 by NCBI. The phenotypic and genotypic characters of the bacterium are summarized in Table 1.

**Table 1:** **Phenotypic and genotypic characters of** ***Serratia marcescens***

**Characters**	**Description**
Colony morphology	Round, smooth, convex (2–5 mm dia.) colonies with red pigment
Microscopic features	single Gram -ve short rods with 0.5-0.8 μm dia and 0.9-2.0 μm length
Biochemical characters	Carbohydrate utilization: +ve for dextrose, fructose, mannose, maltose, mannitol, sucrose and trehalose and –ve for arabinose, galactose, rahmnose, raffinose and melibiose
	Enzyme activity: +ve for catalase and oxidase, −ve for lactose and starch hydrolysis
Physiological characters	Temperature tolerance 4–45°C (opt. 25°C), Produces pigment till 35°C, pH tolerance 3–14 (opt. 5–7), salt tolerance up to 20%
Molecular characters	98.8% similarity with *Serratia marcescens* N4-5
Accessions	Bacterial accession no. MTCC 4822
	Nucleotide sequence no. EF035134

**Figure 1:**
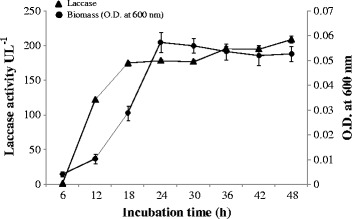
**Laccase activity with corrosponding biomass of** ***Serratia marcescens*** **at 25°C.**

In the growth curve, drawn in laccase medium at an interval of 6 h up to 48 h of incubation, the maximum biomass was attained at 24 h that afterwards showed decline up to 48 h. While the laccase production was recorded maximum at 18 h of incubation that remained almost steady up to 30 h with slight increase during further incubation up to 48 h. An increase in the enzyme production was again recorded at the later stage of stationary phase (48 h) with the highest (208.83 UL^−1^) enzyme titer (Figure 1).

### Partial purification and characterization of laccase

**Figure 2:**
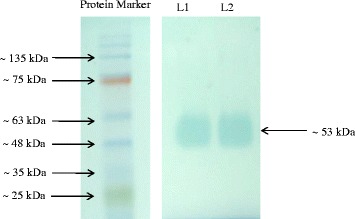
**Native PAGE analysis: Standard protein marker; L1 and L2 contain laccase enzyme with a band of approximate ~ 53 kDa.**

**Figure 3:**
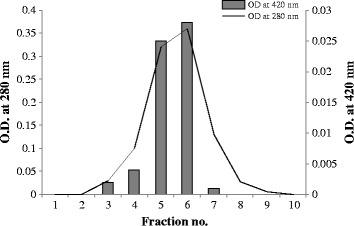
**Elution profile of gel filtration chromatography.**

**Figure 4:**
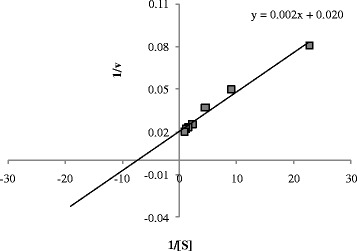
**Line weaver-Burk plot for determination of Km and Vmax of partially purified enzyme.**

Native PAGE revealed the approximate molecular mass of the bacterial laccase 53 kDa (Figure 2). The comparative graph drawn for protein and enzyme with fractions is presented in Figure 3. The enzyme activity was recorded in the fractions 5 and 6. The Km and Vmax value of the partially purified enzyme was 0.10 mM and 50.00 μM min^−1^, respectively, with ABTS. The enzyme activity and protein content estimated at every step of the process is presented in Table 2 and the Lineweaver Burk plot is shown in Figure 4.

**Table 2:** **Partial purification of laccase from** ***Serratia marcescens***

**Sample**	**Volume (ml)**	**Total protein (mg)**	**Total enzyme (Units)**	**Specific activity (Units/mg)**	**Purification**
Crude	200.0	32.00	52.00	1.60	1.00
Acetone precipitated	5.00	1.10	5.50	5.00	3.10
Gel filtration	3.00	0.01	0.08	6.70	4.10

### Effect of the inoculum size, the physico-chemical and nutritional conditions, and the organic solvents on laccase production

#### The inoculum size

**Figure 5:**
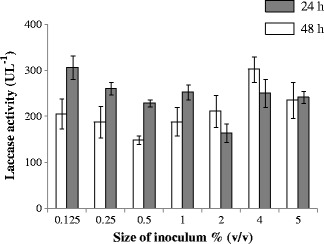
**Effect of inoculum size on laccase production by** ***Serratia marcescens*** **at 25°C.**

The inoculum size of bacterial culture, ranged from 0.125 to 5.00% (v/v), showed significant differences on laccase production (Figure 5). Out of 7 inoculum concentrations used, 4.00% resulted in maximum laccase production, that was significantly higher (P < 0.05) from 2.00% level in two way repeated ANOVA. The effect of bacterial inoculum size also affected the biomass, although the differences were not statistically significant.

#### The physico-chemical and nutritional conditions

**Figure 6:**
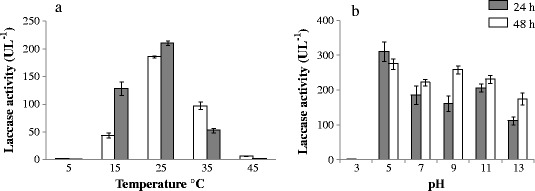
**Effect of physico-chemical factors a) temperature and b) pH on laccase production by** ***Serratia marcescens.***

Among the physico-chemical factors, the incubation temperature and medium pH vitally affected the bacterial milieu conditions and its protein machinery. Between the temperature ranges from 5 to 45°C, the highest production of laccase was recorded at 25°C. Production of laccase at this temperature was significantly higher at P < 0.01 level for all the temperatures, while laccase production at 5 and 45°C were not significantly different (P < 0.05) from each other. From 25°C onward, a steep decline in laccase production was recorded deviating the temperature on either side (<25°C<). While it decreased by 50% at 35°C (P < 0.01), it became negligible at 45°C (Figure 6a). The pH of the medium, considered from 3.0-13.0, also influenced the laccase production significantly (P < 0.05). The enzyme production was recorded highest at pH 5.0 and lowest at pH 3.0 and appeared to be limited in the stationary growth phase. The laccase production at pH 5.0 was significantly higher (P < 0.01) at 24 h in comparison to all the treatments under consideration. The values obtained for pH 5.0 to pH 11.0 at 48 h of incubation were not statistically significant. The enzyme production tended to increase from neutral to pH 13.0, showing significantly higher values (P < 0.05). Variation in production of laccase was influenced by pH with respect to growth phases, the stationary and the exponential growth phase, are shown in Figure 6b. The respective values for biomass, in relation to the temperature and pH, are presented in Table 3.

**Table 3:** **Effect of temperature and pH on bacterial biomass at 24 and 48 h of incubation**

**Temperature (°C)**	**24 h**	**48 h**	**pH**	**24 h**	**48 h**
5	0.014 ± 0.001	0.002 ± 0.001	3	0.004 ± 0.001	0.011 ± 0.002
15	0.024 ± 0.003	0.048 ± 0.003	5	0.069 ± 0.008	0.103 ± 0.012
25	0.058 ± 0.006	0.057 ± 0.001	7	0.057 ± 0.003	0.144 ± 0.008
35	0.082 ± 0.003	0.062 ± 0.007	9	0.061 ± 0.001	0.184 ± 0.013
45	0.053 ± 0.006	0.037 ± 0.004	11	0.081 ± 0.015	0.225 ± 0.024
			13	0.0257 ± 0.061	0.467 ± 0.023

The influence of carbon and nitrogen sources on production of laccase enzyme is summarized in Table 4. All the tested carbon sources showed positive influence on laccase production in basal medium, being statistically highest for xylose (228.4 ± 8.7 UL^−1^) and lowest for glucose (178.3 ± 3.1 UL^−1^) (P < 0.01). However, the enhancement recorded for laccase production in case of xylose was not significantly higher in comparison of the enhancing effect of fructose, maltose and starch (P < 0.01). In case of nitrogen supplementation, organic and inorganic nitrogen sources showed varying responses. Organic sources acted as inhibitors while the sodium nitrate enhanced laccase production in comparison to control (Table 4).

**Table 4:** **Effect of carbon and nitrogen sources on laccase production at 25°C after 24 h of incubation**

**Carbon Source**	**O.D. at 600 nm**	**Laccase activity (UL** ^**−1**^ **)**	**Nitrogen source**	**O.D. at 600 nm**	**Laccase activity (UL** ^**−1**^ **)**
Fructose	0.047 ± 0.005	207.9 ± 3.0	Ammonium Chloride	0.222 ± 0.020	6.0 ± 0.5
Maltose	0.042 ± 0.008	208.4 ± 1.8	Ammonium sulfate	0.135 ± 0.110	3.9 ± 0.4
Sucrose	0.043 ± 0.006	195.1 ± 0.4	Yeast extract	0.912 ± 0.044	17.4 ± 0.5
Xylose	0.048 ± 0.003	228.4 ± 8.7	Casein	0.192 ± 0.023	3.4 ± 0.4
Galactose	0.062 ± 0.008	193.3 ± 4.5	Peptone	0.791 ± 0.074	14.3 ± 0.4
Cellulose	0.037 ± 0.003	200.5 ± 0.5	Urea	0.081 ± 0.031	6.7 ± 0.2
Starch	0.069 ± 0.001	206.6 ± 1.0	Sodium nitrate	0.051 ± 0.004	193.4 ± 6.5

Control: O.D. at 600 nm = 0.040 ± 0.008; Laccase activity = 178.3 ± 3.1 UL^−1^.

#### The organic solvents

Low molecular organic weight solvents behaved as significant enhancers of laccase production in comparison to control during the early phase (24 h) of incubation (P < 0.01). All the solvents enhanced approximately 2–3 folds production at 0.5 to 2.0% concentration. The enhancement declined at later stage (48 h) in case of all the solvents giving values comparable to control, and in case of methanol even being significantly inhibitory (P < 0.05).

## Discussion

Microorganisms are ubiquitous in nature and their distribution is largely governed by environmental specificities. The microbial diversity of IHR is increasingly gaining attention mainly due to their ecological resilience based applications, such as production of secondary metabolites often higher at suboptimal conditions (Mishra et al. 2012; Rinu et al. 2012; Yarzábal 2014). Amongst bacteria, species of *Bacillus*, *Pseudomonas* and *Serratia* have been isolated and studied for their various biotechnological applications (Pandey et al. 1999, 2001, 2006; Malviya et al. 2012; Rinu and Pandey 2009; Selvakumar et al. 2008; Trivedi et al. 2007; Trivedi and Pandey 2008). *Serratia marcescens* MTCC 4822, characterized in the present study for its tolerance to low temperature, wide pH and high salt concentration, has been investigated for laccase production- an enzyme of ecological as well as biotechnological relevance. Absence of pigment production at higher temperature appeared to be in response to particular environmental stimuli that can be considered as a microbial indicator of climate variation. The change in pigment production can be linked to the ability of the bacterial cells to alter their metabolic strategy in response to the environmental conditions. Species of *Serratia* spp are known to give optimum growth from 20-37°C and at pH 7–9 (Giri et al. 2004). *S. marcescens* is characterized by production of a non-diffusible red pigment, named as prodigiosin. The optimum conditions for growth and pigment formation in *S. marcescens* biovar A2/A6 has been reported 30°C in a neutral to slightly alkaline medium (Hardjito et al. 2002).

The bacterial strain produced laccase during its entire growth phase as soon as its metabolism enabled it to outlive the lag phase. Upon entering the exponential phase (6–24 h), the bacterium produced extracellular laccase leading to a steep rise of the laccase activity in the nutrient medium. The growth as well as the enzyme production became steady indicating the onset of stationary phase (24–30 h) of the bacterium, however, it again increased at the later stage of stationary phase. The increase–decrease-increase phenomenon pertaining to the production of laccase can be attributed to the utilization of the nutrients in the medium. The increase in laccase production at the second stage, towards the end of stationary phase, is an indication of the self digestion metabolic activities of the bacterium that has been related to the oxidative properties of the enzyme (Nystrom 2004).

The molecular mass of the laccase from various fungi has been reported between 50 to 140 kDa (Rivera-Hoyos et al. 2013). In a recent case study based on a bacterium (*Micrococcus* sp.) it was found to be 23 kDa (Joseph et al. 2014). The molecular mass of the laccase in the present study was relatively on the higher side (53 kDa). Km values for laccases, in general, have been known ranging between 5 to 500 μM and were recorded 100 μM in the present bacterium. The data on the characterization of laccase, such as molecular mass and Km, provide useful knowledge for further applied studies on protein engineering and phylogeny of the enzyme.

The inoculum size of the bacterium, measured as optical density, affected laccase production. The oxidative catalytic properties of laccase are known to help in self-digestion process of cytoplasmic and outer membranes. The enzyme profile of the bacterium under study was observed to be greatly affected by the final population size and accumulation of end products in the culture medium. Smaller inoculum size (0.125-0.500%) was required for higher enzyme activity in exponential as well as stationary phase. Inoculum size beyond 1% although triggered the enzyme production in stationary phase, it was not sustained at the later stage, i.e., stationary phase. The increase in inoculum size beyond the optimum level is likely to result in rapid depletion of indispensible nutrients. It subsequently may also contribute towards the accumulation of toxic metabolic substances in the growth medium. Similar observations on inoculum size of the bacterium with respect to their metabolic activities have been reported in earlier study (Darah et al. 2013).

Temperature and pH are known to be two of the most important factors affecting the enzyme production. Optimum production of laccase from strains of *Serratia marcescens* has been reported in mesophilic temperature range with neutral to alkaline pH (Chandra et al. 2011; Verma and Madamwar 2003). *Serratia marcescens*, in the present study, exhibited the peculiarity with respect to its ability to produce laccase in a wider temperature (5–45°C) and pH (5–13) range. The extracellular enzymes produced by the microorganisms growing under extreme conditions are known to possess the ability to maintain their activities under harsh conditions contrary to the intracellularly located enzymes which only perform at cytosolic neutral pH. While the ability of the bacterium to produce at wider temperature and pH range is an important feature of ecological relevance for its own survival under harsh conditions, it is also likely to be beneficial for industrial applications such as food, pharmaceutical and textiles. To the best of our knowledge this is the first report on extracellular laccase production by *S. marcescens* isolated from a glacial site under mountain ecosystem of IHR. Albeit, molecular studies on the enzyme under the entire pH scale needs further advance investigation in order to understand the biochemical mechanism of extracellular laccases.

A range of nutritional supplements, mainly the carbon and nitrogen sources, are considered for enhancing the enzyme production from microbial sources. All the carbon sources used in the present study were observed as the enhancers of laccase production probably due to their easy metabolism. In case of nitrogen sources, in-organic and organic sources exhibited variable effects. Two of the inorganic nitrogen sources, sodium nitrate and ammonium nitrate (control), influenced the bacterial biomass in inverse manner in relation to laccase production. The other nitrogen sources promoted growth significantly inhibiting the production of laccase. This indicated the inability of the bacterium to metabolize the nitrogen in form of nitrate, limiting the nitrogen in the medium and indirectly stimulating the laccase expression. Yeast extract, among organic sources, is usually considered as a strong enhancer of laccase production, the inhibitory effect observed due to addition of yeast extract in the present study needs attention in view of the importance of supplements with respect to individual organisms and the specific growth conditions. In general, nitrogen sources are known to regulate the laccase production more effectively in comparison to carbon sources (Madhavi and Lele 2009).

Besides nutritional sources, other supplements are also considered for their effects on laccase production from bacterial sources. Several phenolic and non phenolic compounds have been recognized for the purpose (Muthukumarasamy and Murugan 2014), the use of low molecular weight organic solvents has been rare (Dhakar and Pandey 2013; Lomascolo et al. 2003). In the present study the five organic solvents, selected on the basis of their low molecular weight, found to be efficient enhancers of laccase production. Besides, these organic solvents also possess degradable properties with no harmful effects and are also cost effective.

Extremophiles are becoming the major attraction of the scientists due to their rare and extra ordinary physico-chemical properties. The adaptability of such microorganisms towards broad range of temperature and pH makes them an unbeatable tool for the various purposes in biotechnology. They are likely to be applicable to the processes of biotechnological relevance where the physico-chemical conditions fluctuate frequently and the microorganisms are required to adapt to the fluctuations. Besides the biotechnological relevance, these microorganisms contribute to the geochemical cycles and play crucial role in the dynamics of environmental processes. Combination of peculiar physico-chemical characters, such as tolerance to wide temperature and pH ranges and high salt concentration, along with the production of laccase under these fluctuations, *Serratia marcescens* (MTCC 4822) may serve as a model organism for specific pharmaceutical, biomedical and biotechnological applications.
